# Endophytism: A Multidimensional Approach to Plant–Prokaryotic Microbe Interaction

**DOI:** 10.3389/fmicb.2022.861235

**Published:** 2022-05-12

**Authors:** Simran Rani, Pradeep Kumar, Priyanka Dahiya, Rajat Maheshwari, Amita Suneja Dang, Pooja Suneja

**Affiliations:** ^1^Plant Microbe Interaction Laboratory, Department of Microbiology, Maharshi Dayanand University, Rohtak, India; ^2^Centre for Medical Biotechnology, Maharshi Dayanand University, Rohtak, India

**Keywords:** abiotic stress, biocontrol, detection of PGPEB, endophytism, sustainable agriculture

## Abstract

Plant growth and development are positively regulated by the endophytic microbiome *via* both direct and indirect perspectives. Endophytes use phytohormone production to promote plant health along with other added benefits such as nutrient acquisition, nitrogen fixation, and survival under abiotic and biotic stress conditions. The ability of endophytes to penetrate the plant tissues, reside and interact with the host in multiple ways makes them unique. The common assumption that these endophytes interact with plants in a similar manner as the rhizospheric bacteria is a deterring factor to go deeper into their study, and more focus was on symbiotic associations and plant–pathogen reactions. The current focus has shifted on the complexity of relationships between host plants and their endophytic counterparts. It would be gripping to inspect how endophytes influence host gene expression and can be utilized to climb the ladder of “Sustainable agriculture.” Advancements in various molecular techniques have provided an impetus to elucidate the complexity of endophytic microbiome. The present review is focused on canvassing different aspects concerned with the multidimensional interaction of endophytes with plants along with their application.

## Introduction

Earth has been kneeling under the pressure of a rapidly increasing population which has exerted a lot of stress on the stakeholders, namely, farmers, scientists, and other intermediaries alike. What the world needs right now is extensive, yet a nature-friendly system of agriculture using modern tools along with systems sans application of chemical fertilizers (Lareen et al., 2016). The current system of agriculture is based on the application of chemical fertilizers and other inputs to enhance productivity, thereby leading to destruction of soil nutrients, groundwater contamination, eutrophication, and production of greenhouse gasses, thereby, impacting the overall environment and playing havoc to the health of consumers both humans and animals alike. To overcome this, microbes with plant growth promoting (PGP) traits are being explored to develop potential bioinoculants for sustainable and eco-friendly agriculture.

The plants and microorganisms are well known to interact by various natural amalgams that serve as signaling and nutritive substances for microbes to act upon and influence the nature of the plant microbiome. Plants are naturally associated with microorganisms in rhizosphere, phyllosphere, and endosphere (Dong et al., 2019). The rhizosphere is the tapered region of soil regulated by plant root secretions and associated microbial community termed as root microbiome (Turner et al., 2013; Suneja et al., 2016). Phyllosphere is the microbial habitat on the exterior of above-ground plant organs and the most abundant microbial ecosystem on earth (Vorholt, 2012). Microorganisms living and growing within their host plants are termed endophytes, constituting the plant endosphere. Endophytic bacteria usually complete their life cycle inside host plants without causing any harm to them (Dudeja et al., 2012). However, these bacteria flourish copiously in the rhizosphere due to sufficient nutrition supply by plant root exudates (Canarini et al., 2019).

The abundance of microorganisms in the rhizosphere is known since the beginning of the 20th Century but the endosphere region has not been explored much (Sessitsch et al., 2011). Earlier, the endosphere was known mainly for the fungal group and as a result, our preliminary information about bacterial endophytes remained circumscribed. Various other factors restricted our understanding regarding the action of bacterial endophytes, which includes culturing hitches and lack of pioneering identification techniques. However, endophytic bacteria have attracted a lot of attention since the last two decades owing to recognition of their ability to promote plant growth and their biocontrol potential (Vasileva et al., 2019). This review discusses the PGP endophytic bacteria, their interaction with host plants leading to variations in colonization patterns and diversity, mechanisms of plant growth promotion under normal as well as stress conditions along with omics-led revolution in the field of exploring their bioactive metabolites.

## Plant Growth-Promoting Endophytic Bacteria–Host Plant: Interaction and Colonization

Endophytes (either bacteria or fungi) are defined as colonizers of the internal plant tissues without causing any disease or hostile symptoms and obtained from surface-sterilized tissue of plant (Santoyo et al., 2016; Afzal et al., 2019). Bacterial endophytes are known to be present in every plant part, namely, seeds, rhizomes, roots, nodules, stems, and leaves (Alibrandi et al., 2018). It has been anticipated that endophytic bacteria referred to as the subclass of rhizospheric bacteria or seed-borne bacterial communities, commonly termed as PGP rhizobacteria, established the ability to enter into the host plant (Khare et al., 2018). They possess all vital PGP traits as present in rhizobacteria, but their effect on host plants is typically more significant than rhizobacteria owing to the better adaptation during stress conditions resulting in augmentation of plant growth (Hardoim et al., 2008; Afzal et al., 2019).

The rhizosphere is the interaction point between roots and soil microorganisms (Bulgarelli et al., 2012). Plants release exudates such as organic acids, amino acids, and proteins from their roots, which serve as pre-communication signals between bacterial endophytes and host plants (Kawasaki et al., 2016). Colonization of bacteria into roots occurs through root hairs and, to some extent, through the stem and leaves (Maela, 2019). Some studies have reported that endophytes also colonize through flowers and fruits of the anthosphere and carposphere (Frank et al., 2017). A few regular hotspots have been observed for bacterial colonization such as emergence sites of lateral roots, outer layers of cells, and root cortex (dos Santos et al., 2018). Endophytic bacterial colonization is a multi-stage process that involves (a) chemotactic movement toward roots, (b) root surface attachment, (c) entry inside the root, and (d) movement and localization (Gupta et al., 2012; Kandel et al., 2017a). Table 1 cites various genes involved in the colonization of endophytes.

**TABLE 1:** Genes involved in colonization of endophytes.

Category	Genes	Function	References
Chemotaxis and motility	fliC3	Encodes flagellin	Buschart et al., 2012
	MglB	Galactose chemotaxis	Neumann et al., 2012
	pilX	Type IV fimbrial biogenesis protein PilX	Shidore et al., 2012
	FliI	Flagellar apparatus	Bai et al., 2014; Minamino et al., 2016
	Hsero3720	Methyl accepting chemotaxis transducer transmembrane protein	Balsanelli et al., 2016
	Aer	Aerotaxis	Samanta et al., 2016
	RbsB	Ribose chemotaxis	Reimer et al., 2017
	CheZ	Response regulator	Liu X. et al., 2018; Liu W. et al., 2018
Attachment	lapF gene	Determines biofilm architecture	Martínez-Gil et al., 2010
	gumD	EPS biosynthesis	Meneses et al., 2011
	wssD gene	Cellulose production mutation	Monteiro et al., 2012
	waaL	O-antigen ligase (LPS biosynthesis)	Balsanelli et al., 2013
	eps and tasA	Biofilm formation	Beauregard et al., 2013
	PoaA, PoaB, and PoaC	Lipopeptide	Zachow et al., 2015
	Hsero1294 and fhaB	Filamentous hemagglutinin proteins	Pankievicz et al., 2016
	blr2358	EPS biosynthesis	Xu et al., 2021
Colonization	IacC	IAA degradation necessary for efficient rhizosphere colonization	Zúñiga et al., 2013
	*N*-acyl homoserine lactone Synthase	Quorum Sensing necessary for cell-to-cell communication in efficient colonization	
	EglS	Endo-β-1,4-glucanase (Plant cell wall modification)	Fan et al., 2016

*EPS, exopolysaccharide; LPS, lipopolysaccharide; IAA, indole-3-acetic acid.*

**FIGURE 1:**
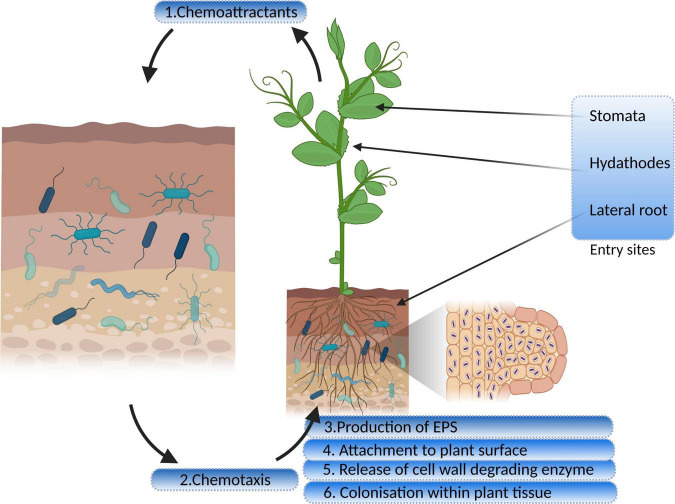
Interaction and colonization of plant growth-promoting endophytic bacteria in the host plant (EPS, exopolysaccharides).

Bacteria, in the vicinity of the roots, receive chemical signals from root exudates and move toward them. Saleh et al. (2020) reported that citric acid, a root exudate of *Brachypodium distachyon*, acting as a strong chemoattractant for PGP bacterial strains. The hypothesis of Streptomyces species being attracted by the root exudates was tested and confirmed by Worsley et al. (2021) on the root exudates of *Arabidopsis thaliana*. The study demonstrated that phytohormone, salicylate, plays a specific role in this process. The genes for proteins encoding motility, chemotaxis, and adhesion are upregulated in response to root exudates, indicating a two-way interaction between the endophyte and its host plant (Jha et al., 2018). Chemotaxis is a significant event in the rhizosphere and the interior parts of roots, for both movement and colonization (Kawasaki et al., 2016). Mutant strains of *Azorhizobium caulinodans* lacking chemotaxis gene cluster (che) were reported to undergo defective colonization owing to its significant role in biofilm formation and exopolysaccharides (EPSs) production (Liu X. et al., 2018; Liu W. et al., 2018, Table 1). Bacterial endophytes primarily bind to the root surface (rhizoplane) and detect the possible entry sites for accessing internal plant tissues (Kandel et al., 2017a). The entry points used by endophytes to reach the host plant are the gaps present in the roots where root hairs or lateral roots arise, as well as the holes in the shoots, wounds, stomata, and hydathodes (Hardoim et al., 2015). Figure 1 illustrates distinct steps of colonization.

Many researchers have stated that extensive bacterial endophyte colonization occurs at the secondary root emergence site. It is because of rapid endophytic penetration at epidermal breakage-point, colonizing at the cortex and subsequent spreading through the endodermis to the vascular tissue (Mahaffee, 1994; Lodewyckx et al., 2002). Endophytes release the cell wall–degrading enzymes such as pectinases, xylanases, cellulases, and endoglucanases before colonizing the roots (Naveed et al., 2013; Maela, 2019). This phenomenon facilitates the entry of bacteria within plant tissues (Kandel et al., 2017a). Various gene sequences have been deduced by comparative genomics, engaged in biofilm formation, adhesion, and motility, leading to plant colonization and maintaining healthy plant–microbe interaction. Bacterial cells synthesize EPSs during the early colonization phase which help the cells to adhere to the root surface. The endophytic strain *Gluconacetobacter diazotrophicus* produces EPS that serves as a critical factor for adhesion and colonization in rice roots (Meneses et al., 2011). The study showed that EPS production by *G. diazotrophicus* shielded the bacterial cells from oxidative damage, and also decreased the concentrations of free radicals. Colonization was found to be reduced in the case of EPS knockout strain of *G. diazotrophicus*, further rescued by the application of wild-type strain (Meneses et al., 2017). Fernández-Llamosas et al. (2021) used homologous recombination for insertional disruption of epsF genes in the genome of *Azoarcus* sp. CIB depicting their role in the efficient colonization of rice roots. Xu et al. (2021) identified EPS biosynthesis gene, blr2358, in *Bradyrhizobium diazoefficiens* USDA110, the mutant of which resulted in a reduced capacity to induce nodules. Other than playing a significant role in plant–endophyte interactions, they exhibit antioxidant, anti-inflammatory, anti-tumor, and prebiotic activities (Liu W. et al., 2018). Lipopolysaccharide machinery is involved in the attachment and proliferation of endophyte colonization that includes the development of flagella and pili, quorum sensing, and movement of bacteria within the host plants (Rocío Suaìrez-Moreno et al., 2010; Scharf et al., 2016). The role of cell wall degrading enzymes in entering and spreading within the host tissue is also very well established. Fan et al. (2016) highlighted the importance of endo-β-1, 4-glucanase in penetration of *Bacillus amyloliquefaciens* into the host tissue. The disruption mutant of eglS gene encoding this enzyme halted colonization; however, overexpression of the same resulted in a substantial increase in the endophyte population. Mechanism of bacterial endophyte attachment with plant surface, entry, survival, is mediated by the cross-talk between host and microorganism, and a lot is to be studied in this regard.

## Endophytism vs. Pathogenicity: Thin Line Between Two Lifestyles

The prevalence of endophytes is decided by chance and genetic indicators of bacteria that promote intermodulation between bacteria and plants, contributing to an active colonization (Dong et al., 2019). Endophytes maintain a smaller cell density to prevent a systemic reaction in comparison to pathogens (Zinniel et al., 2002). They also produce lesser quantities of cell wall degrading enzymes as compared to the phytopathogens that secrete deleteriously large amounts of these enzymes, thereby, preventing the trigger of plant defense systems (Elbeltagy et al., 2000; Afzal et al., 2019). Anabolism-related genes are found to be more diverse and in abundance among the endophytes unlike the phytopathogens having catabolism genes prominently (Hardoim et al., 2015). Endophytes undergo several mechanisms to protect themselves from the plant defense system. Microbe-/pathogen-associated molecular patterns (MAMPs/PAMPs) are the characteristics of microbes recognized by pattern recognition receptors (PRRs) present on the surface of plant cells (Newman et al., 2013). Endophytic bacteria produce MAMPs which either remain unrecognized by plant’s PRRs or induce only a weak reaction as a response in comparison to the pathogenic interactions (Vandenkoornhuyse et al., 2015). They produce enzymes of antioxidant machinery such as superoxide dismutase (SOD), catalase (CAT), peroxidase (POD), glutathione *S*-transferases (GSTs), and alkyl-hydroperoxide reductase C (AhpC), to mitigate the oxidative burst (Zeidler et al., 2004). Bacterial virulence factors are delivered in the extracellular environment or directly into the host by the secretion system (Depluverez et al., 2016). Type-III and Type-VI protein secretion systems, necessary to deliver effector proteins into the plant by pathogens, are altogether absent or present scarcely in endophytic bacteria (Liu W. et al., 2018). Endophytes have been found to undergo a reduction in genome size, which is associated with differences in niche specialization (Lòpez-Fernàndez et al., 2015; Zin et al., 2021). Some bacterial endophytes also downregulate flagella biosynthesis and upregulate functions related to flagellar motor rotation to mask up their flagellin PAMPs and move fast within plants during colonization.

Endophytes have been reported to undergo a change in their lifestyles from endophytes to pathogenic as a result of any imbalance in the host–microbe interaction (Mengistu, 2020). Strategies employed by the plants to distinguish endophytes from pathogens are still a matter of active research. Plett and Martin (2018) have indicated that LysM receptor-like kinases (LysM-RLKs) can differentiate pathogenic signals from those secreted by the mutualistic microbes. It has been suggested that different groups of genes are regulated during colonization in the plants to facilitate the same. The majority of pathways targeted by miRNAs of plant defense system are turned off. These microRNAs otherwise remain stable and can be used as a pathogenicity signal by the plants (Wang et al., 2017). Plants undergo nutrient monitoring to identify parasites and manipulate the ratio of MAMP/DAMP signals to identify the mutualistic signals. However, there are many such receptor/perceptor systems present throughout the plant kingdom that are yet to be studied (Plett and Martin, 2018). Many studies have pointed toward downregulation of plant defense during colonization by mutualistic partners (Khare et al., 2018). In the recruitment of an endophytic companion, the plant host often plays a pivotal role, where the release of specific root exudates and a selective host plant defense response are considered as crucial factors in choosing individual endophytes (Kumar A. et al., 2020).

## Apprehending the Endophytes

**FIGURE 2:**
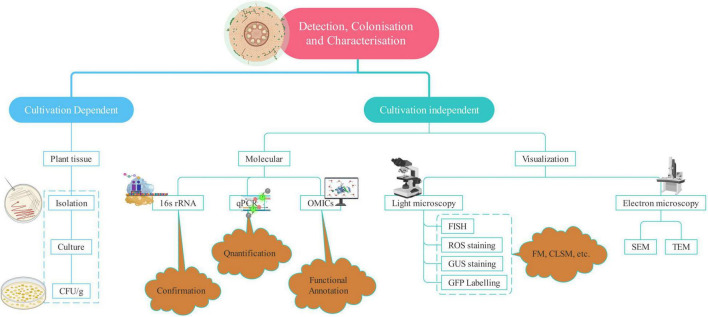
Techniques used for the detection of endophytism.

Enormous benefits provided by endophytes have led to robust research in this field world over. Harnessing their potential to the fullest and large scale application requires a more clear and better understanding of endophytes. It is no less than a challenge as the methods available for detection, isolation, and identification are not sufficient to provide the entire picture of the host–parasite interaction. Cultivation-based studies omit several microbes because it is not possible to reproduce and maintain the optimal conditions required for the growth of most of the microbes (Santoyo et al., 2016). However, the study of endophytes has come a long way from the typical isolation and cultivation methods to more sophisticated ones such as advanced microscopic techniques and “omics”-based studies (Table 2). The amalgamation of two or more techniques helps to significantly increase the discriminatory power of the analysis and a better overview of diversity. Therefore, a combination of techniques is employed to complement each other and to enrich our understanding of the detection and patterns of colonization as shown in Figure 2.

**TABLE 2:** Detection of endophytism.

Technique employed	Endophytes detected	Plant	References
CLSM	*Azotobacter chroococcum* 67B, *Azotobacter chroococcum* 76A	*Solanum lycopersicon*	Viscardi et al., 2016
	*Ralstonia* sp. M1, *Ralstonia* sp. MS1, *Rhizobium* sp. W3, *Rhizobium* sp. SS2, *Rhizobium* sp. R2, *Acinetobacter* sp. M5, *Pantoea* sp. MS3, *Brevundimonas* sp. R3, *Achromobacter* sp. RS1, RS3, RS4, RS5, RS8	*Triticum aestivum*	Patel and Archana, 2017
	*Bacillus cereus* strain XB177	*Solanum melongena*	Achari and Ramesh, 2019
	*Bacillus subtilis* strain 1-L-29	*Camellia oleifera, Arabidopsis thaliana*	Xu et al., 2020
	*Streptomyces* sp. strain SA51	*Solanum lycopersicum*	Vurukonda et al., 2021
	*Bacillus siamensis*	*Cicer arietinum* L.	Gorai et al., 2021
GFP-CLSM-SEM	*Musa*	*Methylobacteriumsalsuginis*	Senthilkumar et al., 2021
FISH	*Arthrobacter agilis* UMCV2 *Bacillus methylotrophicus* M4-96	*Fragaria ananassa*	Hernández-Soberano et al., 2020
FISH-CLSM	*Burkholderia graminis* G2Bd5	*Lolium multiflorum*	Castanheira et al., 2016
	*Gordonia* KMP456-M40, *Enterococcus* KMP789-M107, *Micrococcus* KMP789-MA53, *Staphylococcus* KMP123-MS2, *Staphylococcus* KMP123-MS3, *Acinetobacter* KMP123-MA14, *Bacillus* KMP123-MS1	Mangroves	Soldan et al., 2019
	Firmicutes, Gammaproteobacteria	*Citrus limon*	Faddetta et al., 2021
DOPE-FISH-CLSM	*Streptomyces mutabilis*	*Triticum aestivum*	Toumatia et al., 2016
FISH-GFP-CLSM	*Pseudomonas* G1Dc10, *Paenibacillus* G3Ac9, *Sphingomonas azotifigens* DSMZ 18530	*Lolium multiflorum*	Castanheira et al., 2017
DOPE-FISH-CLSM-SEM	Alphaproteobacteria, Betaproteobacteria, Gammaproteobacteria, Firmicutes, and Actinobacteria	*Cucumis melo reticulates group* cv. ‘Dulce’	Glassner et al., 2018
Fluorescence microscopy	Diazotrophic endophytes	*Oryza sativa*	Kandel et al., 2015
ROS staining combined with Light microscopy	*Burkholderia gladioli*	*Panicum virgatum*	White et al., 2014
	*Enterobacter cloacae*	*Agave tequilana*	Lima et al., 2018
	*B. amyloliquefaciens*	*Gossypium*	Irizarry and White, 2018
	LMT2b (*Microbacterium* sp.), LMY1a (*Pseudomonas baetica*), LTE3 (*Pantoea hericii*), LTE8 (*Paenibacillus* sp.), LYE4a (*Pseudomonas oryzihabitans*), LYY2b (*Pantoea vagans*), LLE3a (*P. agglomerans*)	*Oryza sativa* L., *Cynodon dactylon* L.	Verma et al., 2018
	*Pseudomonas* sp., *Bacillus* sp., *Paenibacillus* sp., *Microbacterium* sp., *Exiguobacterium* sp.	*Triticum aestivum*	Patel et al., 2021
SEM	*Azospirillum* spp., *Azoarcus* spp., *Azorhizobium* spp.	*Triticum aestivum* L.	Dal Cortivo et al., 2017
TEM	*Azotobacter chroococcum*	*Arnebia hispidissima*	Singh and Sharma, 2016
	*Bacillus subtilis* and *Serratia marcescens*	*Centella asiatica*	War Nongkhlaw and Joshi, 2017
	*Bacillus methylotrophicus*	*Potentilla fulgens*	
	*Bacillus* sp.	*Houttuynia cordata*	
SEM TEM	*Enterobacter hormaeche* RCE1, *Enterobacter asberiae* RCE2, *Enterobacter ludwigii* RCE5, *Klebsiella pneumoniae* RCE7	*Citrus reticulate*	Thokchom et al., 2017
GFP-SEM-TEM-Real Time RT-PCR	*Bacillus amyloliquefaciens*	*Zea mays, Arabidopsis thaliana* and *Lemna minor*	Fan et al., 2011
PCR-DGGE	*Burkholderia* sp. J62 *Pseudomonas thivervalensis* Y-1-3-9	Rape plants	Chen et al., 2013
FRET	*A. chroococcum* Avi2 strain	*Oryza sativa*	Banik et al., 2016
Serial dilution plating-CLSM-Bio-PCR	*Pseudomonas putida* BP25 (PpBP25)	*Arabidopsis thaliana*	Sheoran et al., 2016
Light microscopy-TEM-SEM-Qpcr	*Shinella* sp. UYSO24 and *Enterobacter* sp. UYSO10	*Saccharum officinarum*	Taulé et al., 2016
Real Time PCR	*Pseudomonas putida*	*Piper nigrum* L.	Agisha et al., 2017

*GFP, green fluorescent protein; CLSM, confocal laser scanning microscopy; SEM, scanning electron microscopy; FISH, fluorescence in situ hybridization; DOPE-FISH, double labeling of oligonucleotide probes for FISH; ROS, reactive oxygen species; TEM, transmission electron microscopy; Real time RT-PCR, real time reverse transcriptase polymerase chain reaction; PCR-DGGE, polymerase chain reaction denaturing gradient gel electrophoresis; FRET, fluorescence resonance energy transfer.*

Microscopy is the sole direct method to observe the endophytes, which helps in understanding the mode of infection, tissue-specific concentration, and the extent of colonization along with the plant response (Johnston et al., 2006). Both light and electron microscopy can reveal the exact location of endophytes within the plant tissue (Kuldau and Yates, 2000). Electron microscopy provides us with the ultrastructural analysis of the endophytes (Pimentel Esposito-Polesi et al., 2017). Transmission electron microscopy (TEM) and scanning electron microscopy (SEM) yield valuable information about the inner structure and surface of the sample, respectively (Ramanujam et al., 2016). Electron microscopy has provided a lot of assistance in the detection of endophytes, the extent of colonization, interaction with the host, and establishment within the plant environment (War Nongkhlaw and Joshi, 2017). Fluorescence *in situ* hybridization is a powerful technique to analyze the microorganisms and screen various microbial communities employing group-specific probes. It involves targeting 16S rRNA gene’s conserved region or species-specific probe to observe the individual cells of endophytic bacteria (Castanheira et al., 2017). Green fluorescent protein (GFP) tagging and β-glucuronidase (GUS) staining rely on the broad host plasmids containing constitutively expressed GUS or GFP genes for tracking bacteria within the endosphere (Robertson-Albertyn et al., 2017). GFP-tagged endophytes fluoresce in the presence of UV or blue light and oxygen thus unfolding the information concerned with the success of colonization as well as the sites of entry (Reinhold-Hurek and Hurek, 2011).

Reactive oxygen species (ROS) staining is a more convenient and cost-effective method than the visualization techniques, for instance, TEM and Fluorescent microscopy. Similar to ROS, hydrogen peroxide (H_2_O_2_) and superoxide (O_2_^–^) radicals associated with the microbial invasion of eukaryotic cells are stained by 3,3′-diaminobenzidine tetrachloride (DAB) and nitroblue tetrazolium (NBT), respectively. DAB results in brown coloration, indicating the presence of endophytes in tissues (White et al., 2014). NBT stains superoxide radicals, which reduce NBT and it results in dark blue and water-insoluble formazan (Shinogi et al., 2003). It is used to detect endophytes in different plant tissues unlike DAB, which is not able to detect endophytes in shoot tissues because of the inability of DAB to penetrate the same.

Genomics-based studies such as next-generation sequencing and continued development in bioinformatics have allowed a significant improvement in our understanding of plant–endophyte interactions. Metagenomics and transcriptomics in particular are proving to be extremely useful for analyzing the functional characteristics of endophyte species (Kaul et al., 2016). Molecular methods are being readily employed to cultivate bacteria for their identification and enable to distinguish the bacterial populations in plant tissues (Afzal et al., 2019). These have allowed a more rigorous analysis of endophytic bacteria’s abundance and community composition (Santoyo et al., 2016). Different molecular methods used to characterize the endophytic bacteria include 16S rDNA sequencing, randomly amplified polymorphic DNA (RAPD), BOX-A_1_R-based repetitive extragenic palindromic PCR, amplified ribosomal DNA restriction analysis, enterobacterial repetitive intergeneric consensus, denaturing gradient gel electrophoresis (PCR-DGGE) and repetitive extra-genic palindromic sequence.

Ten bacterial endophytes were isolated and identified from three different cereals, *Triticum aestivum, Oryza sativa*, and *Zea mays* (Liu et al., 2017a). These isolates were classified by 879F-RAPD and 16S rDNA sequencing followed by clustering into seven groups signifying a clonal origin and assigned into four genera, *Paenibacillus, Enterobacter, Pantoea*, and *Klebsiella*. Recently, Zheng et al. (2020) identified root nodule endophytes from *Sesbania cannabina* and *Glycine soja* using PacBio’s circular consensus sequencing of full-length 16S rDNA gene for more accurate taxonomic information. These nodule isolates were assigned to 18 genera and 55 species, *Ensifer* being the predominant genera. PacBio technology helps in less ambiguous classification and provides finer taxonomic details. This technique has also recently been used to explore microbial communities in different samples (Singer et al., 2016; Motooka et al., 2017; Pootakham et al., 2017). Lastochkina et al. (2020) performed RAPD-PCR analysis to confirm the identity of *Bacillus subtilis* within the internal tissues of *T. aestivum* L. The endophytic diversity and detailed analysis of endophytic bacterial composition from the commercial crop (*Paullinia cupana*) of Brazil using PCR-DGGE were studied. The study disclosed the presence of phyla Firmicutes, Proteobacteria, Actinobacteria, Bacteroidetes, and Acidobacteria, Firmicutes being the predominant phylum (Bogas et al., 2015). The endophytic community of *Distichlis spicata*, *Plucheaab sinthiodes*, *Gaultheria mucronate*, and *Hieracium pilosella* growing in extreme environments of Chile (Atacama desert and Patagonia) was studied. The composition and diversity were analyzed using quantitative PCR and high-throughput gene sequencing of 16S rDNA. The endophytes from both the regions were categorized into phylum Proteobacteria, Firmicutes, Actinobacteria, and Bacteroidetes (Zhang et al., 2019). Upon extensive data mining of endophytic diversity from various plants, it has been observed that the members of phylum Proteobacteria, Actinobacteria, and Firmicutes were the most dominant (Rana et al., 2020; Bhutani et al., 2021).

## Plant Growth Promoting Endophytic Bacteria: The Base of “Sustainable Agroecosystem”

Plant growth promoting endophytic bacteria (PGPEB) are well known to enhance the growth of plants directly and indirectly. They benefit directly to host by the concerted activity of biological nitrogen fixation, phytohormones production, phosphate solubilization, and modulation of 1-aminocyclopropane-1-carboxylic acid (ACC) deaminase expression for better growth under normal and stress conditions. Endophytes being in direct association with plants provide nitrogen in the functional form to their host either by fixing atmospheric nitrogen or by producing ammonia (Marques et al., 2010; Brígido et al., 2019). The frequent usage of nitrogen in the form of chemical fertilizers predominantly increases the cost of crop production. Hence, ammonia production by bacterial endophytes is an essential attribute for the selection of desirable bioinoculants (Li et al., 2017).

Solubilization and mineralization of phosphate are accomplished by bacterial endophytes, which assist in lowering the pH by releasing various organic acids that break Ca-bonded phosphorus in the bound form of soil (Sharma et al., 2013). Members of several genera have been reported as efficient phosphate solubilizers, such as *Rhizobium, Bacillus, Serratia, Arthrobacter, Burkholderia, Pseudomonas, Erwinia*, and *Microbacterium* (Oteino et al., 2015; Li et al., 2017; Srinivasan et al., 2018). The production of phytohormones by PGPEB is another mechanism that significantly boosts the growth of plants and alters the plant morphology (de Souza et al., 2015). IAA is a commonly produced auxin by endophytic bacteria that controls various growth processes in plants, including cell division, elongation, differentiation, gravity, and light responses (Bhutani et al., 2018a). Thus, it aids the host plant in nutrient absorption (Dhungana and Itoh, 2019). There have been numerous studies documenting IAA-producing endophytic bacteria in addition to endogenous IAA in plants (Khan and Doty, 2009; Mohite, 2013; Etesami et al., 2015; Bhutani et al., 2018b; Maheshwari et al., 2021). The application of IAA-producing bacteria to plants has demonstrated substantial upsurge in growth and yield. In addition to this, microbial IAA has been reported as a signaling molecule in several interactions between plants and microbes (Duca et al., 2014).

Gaseous phytohormone, ethylene and its precursor 1-aminocyclopropane 1-carboxylic acid (ACC), play a significant role in response to a wide range of stresses (Glick, 2014). Symbiotic bacteria ease the negative impact of ethylene on plants by expressing the ACC deaminase (ACCD). A variety of endophytic bacteria such as *Azospirillum, Ralstonia, Pseudomonas, Rhizobium, Agrobacterium, Enterobacter, Achromobacter*, and *Bacillus mojavensis* possess ACC deaminase gene and are characterized for ACCD activity (Blaha et al., 2006; Glick et al., 2007; Maheshwari et al., 2020). Transgenic varieties of plants have been developed which, by expressing the bacterial ACCD gene, have improved stress tolerance mechanisms (Stearns and Glick, 2003; Singh et al., 2015). But the addition of ACCD-producing endophytic bacteria could be more cost-effective, readily available, and environmentally sustainable, with higher acceptability compared to transgenics (Glick, 2012; Barnawal et al., 2016).

Plant growth-promoting endophytic bacteria–based biofertilizers can be envisioned as the future nutrient delivery system for plants. This approach, if carried out effectively, can bring about “real” green revolution, which will be more sustainable and reliable (Lugtenberg and Kamilova, 2009; Liu et al., 2017b). To develop these endophytes as microbial inoculants in agriculture, their functional characterization based on PGP traits and *in vivo* evaluation to check their efficacy should be the first prerequisite (de Souza et al., 2015; Alori and Babalola, 2018). Ferchichi et al. (2019) evaluated *in vitro* and *in vivo* efficacy of bacterial endophytes from three species of *Lupinus*. Two endophytes *Paenibacillus glycanilyticus* and *Pseudomonas brenneri* possessed desired functional characters and promoted plant growth *in vivo* and *in vitro* that can be developed as eco-friendly biofertilizers to boost up *Lupinus* productivity. Recently, the multifunctional potential of *Paenibacillus polymyxa* isolated from bulbs of *Lilium lancifolium* was assessed (Khan M. S. et al., 2020). The bacterial isolate possessed various PGP traits, including the production of IAA, siderophores, ACCD, fixation of nitrogen, and solubilization of phosphate. It also promoted the plant growth of different *Lilium* varieties under greenhouse conditions. The study demonstrated that the potential of *P. polymyxa* can be evaluated as an effective bioinoculant. Before any *ex-planta* application for the agricultural crop, some pre-requisites need to be fulfilled for selection as an inoculant. These factors include colonization ability in plant roots, competition with other microflora and their survival in soil, upsurge exudate production which acts as a bridge between plant and bacteria, and improvement of soil health (Beauregard et al., 2013; Carvalhais et al., 2013; Ríos-Ruiz et al., 2019). Seven bacterial endophytes were isolated from different legume crops, namely, *Glycine max, Vigna unguiculata, Vigna mungo, Vigna radiata*, and *Arachis hypogaea* and categorized on the basis of PGP traits. These isolates were used to bacterize the seeds of *A. hypogaea* for plant growth stimulation experiments using gnotobiotic systems and in pots. The results depicted a positive influence on *A. hypogaea* growth. Additional treatment given along with chemical fertilizer at 50% recommended dose, positively affected *A. hypogaea* growth, but the negative effect was seen over the bacterial population when the dose of fertilizers exceeded more than 50%. Their results suggested that root nodules harbor the endophytic population, which augments the growth of a plant, and the addition of fertilizers adversely affects their population and activity (Dhole et al., 2016). The 28 bacterial endophytes were isolated from dry and germinating seeds of *Cicer arietinum* and characterized for PGP attributes. Molecular identification analysis showed that these endophytes belong to *Pseudomonas* sp., *Enterobacter* sp., *Bacillus* sp., *Mixta* sp., and *Pantoea* sp. These were applied to *C. arietinum* roots and led to an increase in plant length, biomass, and chlorophyll content along with biocontrol activity against *Fusarium oxysporum* (Mukherjee et al., 2020). Maheshwari et al. (2019a) isolated and investigated endophytic bacteria from *C. arietinum* and *Pisum sativum* for PGP attributes. Most efficient isolates were identified as *Pantoea agglomerans*, *Bacillus cereus*, *Bacillus sonorensis*, *Bacillus subtilis*, *Pseudomonas chlororaphis*, *Ornithibacillus* sp., and *Ochrobactrum* sp. These studies convey direct evidence for the occurrence of valuable endophytes, which can be further harnessed as bioinoculants for improving plant health.

## Inducing Climate Change Resilience in Flora

Plant community throughout the world is suffering in terms of growth, development, and yield as a result of climate change–induced environmental stress manifested in the form of drought, flood, temperature extremes, salinity, heavy metal toxicity combined with biotic stresses caused due to herbivores, pathogens, and so on. Enough studies have reported the significant role played by endophytes in mitigating abiotic and biotic stress (Rani et al., 2021). Different mechanisms have been deciphered revealing the complex regulation involved in the stress tolerance conferred by the endophytes to the host plants. Drought, salinity, and temperature extremes have been reported as the most devastating abiotic stresses for crops as far as the yield is concerned. Plants respond to drought and salinity *via* stomatal closure, reduced turgidity, osmotic stress, ultimately reducing growth and yield (Van Zelm et al., 2020). Increased evaporation induced by high temperature leads to water loss resulting in the formation of protein aggregates as a result of protein folding inhibition, whereas low temperature leads to the formation of ice crystals causing permanent damage to cells (Lamers et al., 2020). Extreme temperature alters membrane fluidity (Nicolson, 2014).

Endophytes alleviate these stresses by upregulating aquaporins, improving the level of abscisic acid, ACCD activity, enhancing enzymatic and non-enzymatic ROS scavenging machinery and osmolytes, higher expression of ion channels KAT1 and KAT2 resulting in decreased Na^+^/K^+^, adjusting gene expression, and reducing malondialdehyde (MDA) content (Cohen et al., 2015; Gond et al., 2015; Barnawal et al., 2016; Abdelaziz et al., 2017; Xu et al., 2017; Zhang et al., 2017; Sapre et al., 2018; Wang et al., 2018). Likewise, toxic metal ions evoke oxidative stress by generating ROS which promote DNA damage/impairment of DNA repair mechanisms, membrane functional integrity, nutrient homeostasis, and perturb protein function and activity (Tamás et al., 2014). Endophytes induce heavy metal tolerance to their hosts by reducing the mobility of heavy metals by chelation or intracellular sequestration and limiting the translocation of heavy metal ions from roots to shoots (Leonhardt et al., 2014). Table 3 describes various endophytes conferring abiotic stress protection to their hosts by different mechanisms.

**TABLE 3:** Endophytes inducing abiotic stress tolerance in host plants.

Stress tolerance	Host	Endophytes	Mechanism of action	References
Drought	*Arabidopsis thaliana*	*Azospirillum brasilense*	Enhancement of ABA	Cohen et al., 2015
	*Populus deltoids*	*Rhodotorula graminis, Burkholderia vietnamiensis*, and *Rhizobium tropici*	Host plant damage reduced by ROS scavenging machinery	Khan A. et al., 2016
	*Oryza sativa*	*Piriformospora indica*	Regulation of miR159/miR396 that target MYB and GRF transcription factors involved in regulation of growth and hyposensitivity response	Fard et al., 2017
	*Zea mays* L.	*Piriformospora indica*	Enhanced antioxidant enzyme activity, proline accumulation, and expression of drought-related genes and lowered membrane damage	Xu et al., 2017
	*Elymus dahuricus* and *Triticum aestivum*	*Alternaria alternata* LQ1230	IAA secretion contributes to the growth and upregulation of antioxidant enzymes activities and osmoregulatory substances	Qiang et al., 2019
	*Hordeum vulgare*	*Piriformospora indica*	Resources in host redistributed to reduce negative impact of stress and presence of aquaporin water channels sustained	Ghaffari et al., 2019
Salinity	*Lycopersicon esculentum*	*Pseudomonas fluorescens* and *Pseudomonas migulae*	ACC deaminase activity	Ali et al., 2014
	*Chlorophytum borivilianum*	*Brachybacterium paraconglomeratum* strain SMR20	Potential deamination of ACC in the host roots leading to decreased production of stress ethylene, delayed chlorosis and senescence that resulted in improved yield of plants	Barnawal et al., 2016
	*Triticum aestivum*	*Dietzia natronolimnaea*	Enhanced expression of TaST, a salt stress-induced gene	Bharti et al., 2016
	*Oryza sativa*	*Bacillus pumilus*	Effective salt tolerance, survivability, root colonization and multifarious PGP trait, significant reduction in antioxidant enzyme activities and MDA content	Khan Z. et al., 2016
	*Zea mays*	*Pseudomonas fluorescens* 002	Release of IAA and protection of plants against the inhibitory effects of NaCl	Zerrouk et al., 2016
	*Triticum aestivum*	*Arthrobacter protophormiae* SA3, *Dietzia natronolimnaea* STR1, and *Bacillus subtilis* LDR2	IAA content of wheat increased under salt and drought stress conditions. SA3 and LDR2 inoculation counteracted increase of ABA and ACC	Barnawal et al., 2017
	*Triticum aestivum*	*Chryseobacterium gleum* sp. SUK	Improved root-shoot length, fresh-dry weight, chlorophyll, proteins, amino acids, phenolics, flavonoids content and decreased level of proline, Na^+^ uptake, increase in K^+^ uptake	Bhise et al., 2017
	*Cicer arietinum*	*Mesorhizobium ciceri* and *Bacillus subtilis*	Decreased H_2_O_2_ concentration and improved proline contents.	Egamberdieva et al., 2017
	*Avena sativa*	*Klebsiella* sp.	Biochemical parameters such as proline content, electrolyte leakage, MDA content and antioxidant enzyme activities analyzed and found to be notably lesser in IG3 inoculated plants	Sapre et al., 2018
	*Oryza sativa*	*Burkholderia* strain P50	ACC deaminase activity and united PGP traits of P50 successfully alleviate salt stress in rice seedlings by improving morphological and biochemical parameters and decreasing ROS scavenging antioxidant enzymes, osmolytes and stress ethylene	Sarkar et al., 2018
	*Capsicum annuum* L.	*Bacillus* sp.	Induced high levels of proline production and antioxidant enzyme activities	Wang et al., 2018
	*Oryza sativa*	*Curtobacterium albidum* strain SRV4	SRV4 expressed positive attribute for nitrogen fixation, EPS, HCN, IAA, and ACCD activity leading to improvement in plant growth parameters, photosynthetic efficiency, membrane stabilization index and proline content, antioxidative enzymatic activities and K^+^ uptake	Vimal et al., 2019
Heat	*Lycopersicon esculentum* Mill	*Paraburkholderia phytofirmans*	Accumulation of sugars, total amino acids, proline, and malate, promotion of gas exchange	Issa et al., 2018
	*Glycine max*	*Bacillus cereus* SA1	Induction in the endogenous levels of several phytohormones (ABA and SA), essential amino acids	Khan M. A. et al., 2020
Cold	*Arabidopsis thaliana*	*Burkholderia phytofirmans* strain PsJN	Significant changes in PS-II activity, differential accumulation of pigments	Su et al., 2015
	*Solanum lycopersicum* Mill.	*Pseudomonas vancouverensis* and *P. frederiksbergensis*	Improved reactive oxygen species levels and reduced membrane damage and high expression of cold acclimation genes LeCBF1 and LeCBF3	Subramanian et al., 2015
	*Lycopersicon esculentum*	*Bacillus cereus; Bacillus subtilis; Serratia* sp.	Promoting soluble sugar, proline, and osmotin accumulation, enhancing antioxidant defense system	Wang et al., 2016
Heavy metal	*Miscanthus sinensis*	*Pseudomonas koreensis* AGB-1	High tolerance to Zn, Cd, As, and Pb by extracellular sequestration, increased CAT and SOD activities	Babu et al., 2015
	*Solanum nigrum*	*Pseudomonas aeruginosa*	Enhanced Cd stress tolerance	Shi et al., 2016
	*Panicum virgatum* L.	*Pseudomonas putida* Bj05, *Pseudomonas fluorescens* Ps14, *Enterobacter* spp. Le14, So02, and Bo03	Plants protected from inhibitory effects of Cd, plant growth improved and Cd concentration decreased	Afzal S. et al., 2017
	*Zea mays* L.	*Gaeumannomyces cylindrosporus*	Height, basal diameter, root length, and biomass of maize seedlings increased significantly under Pb stress	Ban et al., 2017
	*Glycine max* L.	*Sphingomonas* sp.	Reduced Cr translocation to roots, shoot, and leaves and oxidative stress was significantly reduced regulating reduced GSH and enzymatic antioxidant CAT	Bilal et al., 2018
	*Oryza sativa*	*Enterobacter ludwigii* SAK5, *Exiguobacterium indicum* SA22	Protection against heavy metal Cd and Ni hyperaccumulation by enhanced detoxification mechanisms	Jan et al., 2019
	*Brassica juncea*	*Serratia* sp., *Enterobacter* sp.	Phytohormone production, phosphate solubilization, and antioxidative support responsible for Cd resistance	Ullah et al., 2019
	*Saccharum officinarum*	*Pseudomonas fluorescens*, *Kosakonia radicincitans*, *Paraburkholderia tropica*, and *Herbaspirillum frisingense*	Alleviating Al stress	Labanca et al., 2020
	*Brassica napus* L.	*Serratia* sp. IU01	Minimized the magnitude of the oxidative damage and advantages in terms of growth promotion and alleviating Cd toxicity	Shah et al., 2020

*ABA, abscisic acid; MYB, myeloblastosis family; GRF, growth-regulating factors; ACCD, 1-aminocyclopropane-1-carboxylic acid deaminase; MDA, malondialdehyde; HCN, hydrogen cyanide; SA, salicylic acid; CAT, catalase; SOD, superoxide dismutase; GSH, glutathione.*

## Plant Growth Promoting Endophytic Bacteria-Assisted Biocontrol of Phytopathogens

**FIGURE 3:**
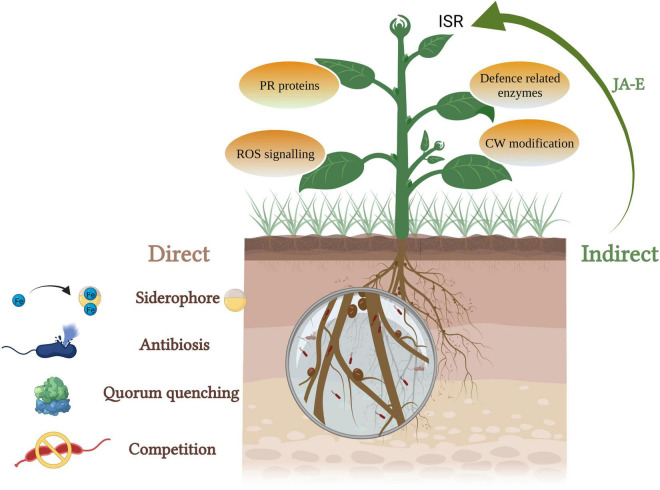
PGPEB-mediated direct and indirect biocontrol of phytopathogens (JA, jasmonic acid; E, ethylene; ISR, induced systemic resistance; PR, pathogenesis related; ROS, reactive oxygen species; CW, cell wall).

Several microbes (viruses, bacteria, and fungi), nematodes, and insects are responsible for infecting plants leading to biotic stress. Physical barriers such as cuticle, wax, trichomes, etc., form first line of defense for the plants (Iqbal et al., 2021). Promoting the availability and absorption of nutrients, augmentation of stress tolerance and disease resistance of disease are the key means of plant disease control by endophytic bacteria (Hamilton et al., 2012). The most commonly reported bacterial genera with biocontrol activity are *Bacillus, Actinobacteria, Enterobacter, Pseudomonas, Paenibacillus*, and *Serratia* (Ek-Ramos et al., 2019). These mechanisms can be broadly grouped into direct and indirect biocontrol activities (Figure 3).

Direct biocontrol involves the production of growth-inhibiting compounds such as siderophores, hydrogen cyanide, cell wall–degrading enzymes, and quorum-sensing inhibitors. Siderophore complex provides Fe to plants during the scarcity and assists them in iron acquisition (Dimkpa, 2016). A lot of work has been done to screen siderophore-producing endophytic bacteria–mediated improvement in plant growth along with biocontrol of phytopathogens (Lacava et al., 2008; Pandey et al., 2015; Passari et al., 2016; Walitang et al., 2017; Yan et al., 2018; Maheshwari et al., 2019b). A number of studies have demonstrated that siderophore-producing bacteria support the plants to endure both biotic and abiotic stresses. In a study by Ruiz et al. (2015), *Pseudomonas protegens* strain survived in the presence of Fusaric acid (mycotoxin)-producing *Fusarium* strains by producing metal scavenging siderophores (pyoverdine and pyochelin). Recently, Butaite et al. (2017) found that non- and low-pyoverdine siderophore producers coexist in various natural populations. The non-siderophore producers with suitable siderophore receptors can utilize external siderophores. The producers are different in the types of pyoverdine they secrete and offer protection against exploitation by non-producers and acquisition of iron unaccessible to opposing strains lacking the proper receptors.

Hydrogen cyanide (HCN) production leads to the growth inhibition of pathogens due to biogenic cyanogenesis. Cyanide, a metabolic inhibitor, inhibits the cytochrome C oxidase and the other metalloenzymes of the pathogen and thus helps the plant to combat against soil-borne diseases (Voisard et al., 1989; Blumer and Haas, 2000; Dheeman et al., 2019; Maheshwari et al., 2019a; Swarnalakshmi et al., 2019). Dubey et al. (2014) reported *Bacillus subtilis* strain producing HCN and other metabolites that inhibit the growth of phytopathogen *Fusarium oxysporum*. HCN-producing endophytes isolated from *Glycine max* exhibited *in vitro* antagonism against a wide range of phytopathogens, namely, *Sclerotium rolfsii, Rhizoctonia solani, Alternaria alternata, F. oxysporum, Macrophomina phaseolina*, and *Colletotrichum truncatum* (Dalal et al., 2015). The bacterial endophytes obtained from the medicinal plant *Clerodendrum colebrookianum* Walp possessed *in vitro* PGP activities including HCN production. These endophytes exhibited *in vitro* and *in vivo* biocontrol activity against various phytopathogenic fungi (Passari et al., 2016). Quorum sensing is mediated by small diffusible signaling molecules called autoinducers which mediate the regulation of diverse functions such as virulence and biofilm formation. Endophytic bacteria can interfere in quorum sensing using quorum sensing inhibitors and quorum quenching enzymes to control bacterial infections as a result of suppressing the formation of biofilm (Zhou et al., 2020). Many endophytic bacteria have been reported to produce lactonases and acylases which results in quorum quenching by the inactivation/degradation of major signaling molecules involved in quorum sensing, *N*-acyl homoserine lactones (Rashid et al., 2012).

Indirectly, endophytic bacteria are known to trigger jasmonic acid and ethylene-mediated induced systemic resistance which induces a defense mechanism and protects the plants from future attacks of plant pathogens (Miliute et al., 2015). It leads to the production of pathogenesis-related proteins, phytoalexins, defense-related enzymes such as polyphenol oxidase (PPO) and phenylalanine ammonia lyase, formation of physical barriers such as cuticles and modification of cell wall (Wiesel et al., 2014). Mao et al. (2019) demonstrated endophytic bacterial strain REB01 to induce disease resistance against rice sheath blight caused by *R. solani via* enhancing the activity of PPO, POD enzymes, and reducing the MDA content. Kim et al. (2019) isolated and selected pine endophytic bacteria on the basis of the relative expression of defense-related genes, *Pseudomonas putida* 16 YSM-E48, *Curtobacterium pusillum* 16YSM-P180, and *Stenotrophomonas rhizophila* 16YSM-P3G, effective against *Bursaphelenchus xylophilus* (pinewood nematode) causing pine wilt disease. Asghari et al. (2020) reported induction of systemic resistance in *Vitis vinifera*, by *Pseudomonas* sp. Sn48 and *Pantoea* sp. Sa14 against *Agrobacterium tumefaciens* through improvement in the levels of PR1, PR2, and PR4 gene expression levels of plantlets.

Some of the endophytic bacteria undergo rhizophagy, biphasic cycle of alternation between the root intracellular phase (nutrients extracted by plants) and a free-living soil phase (acquisition of nutrients by bacteria) (Dudeja et al., 2021). “Endobiome interference” is the term used to describe the phenomenon in which other endophytes interfere with rhizophagy and extract the nutrients from native microbes post-colonization. Although the mechanism behind this remains poorly understood, it has been hypothesized that the oxidative resistance of microbes reduces the capacity of host cells to control the intracellular microbes using ROS produced by NOX enzymes on the root cell plasma membranes. They can be explored to develop bioherbicides to target competitive weeds (White et al., 2019). This way they enhance stress in the host and inhibit their growth leading to an eco-friendly biocontrol option (Verma et al., 2021). Kowalski et al. (2018) explored endobiome interference by the application of a bacterial endophyte, *Micrococcus luteus*, isolated from the seedling root hairs of *Lycopersicon esculentum*, to arrest the growth of a weedy plant sp., *Phragmites australis*, by targeting its growth promotional native microbiome. It also illustrates a vital precaution to be taken before applying any exogenous endophytes, that is, to analyze the interactions between the endophytes being applied and the native microflora (Verma et al., 2021). Table 4 describes numerous studies depicting the biocontrol potential of the bacterial endophytes.

**TABLE 4:** Biocontrol of phytopathogens using bacterial endophytes.

Host Plant	Endophytes	Disease	Causing agent	Mechanism	References
**Diseases caused by fungi**					
*Zea mays* L.	*Bacillus amyloliquefaciens* subsp. *subtilis*	Ear rot and stalk rot	*Fusarium moniliforme*	PR-1, PR-10 genes highly induced	Gond et al., 2015
*Nicotiana glauca*	*Alcaligenes faecalis* S18, *Bacillus cereus* S42	Fusarium wilt	*Fusarium oxysporum* f. sp. *lycopersici*	Proteolytic and chitinolytic activity, HCN production	Aydi Ben Abdallah et al., 2016
Salicaceae plants	*Burkholderia* strains WP40 and WP42	Root rot, Ear blight or scab, Take all, Seed blight or rot	*Rhizoctonia solani* AG-8, *Fusarium culmorum, Gaeumannomyces graminis* var. *tritici, Pythium ultimum*	Production of HCN and antifungal metabolite, occidofungin	Kandel et al., 2017b
*Dodonaea viscosa* L.	*Bacillus, Pseudomonas, and Streptomyces*	Black mold, Fusarium wilt	*Aspergillus niger, Fusarium oxysporum*	Chitinase, protease and antifungal activity	Afzal I. et al., 2017
*Fragaria* × *ananassa* (Duch.)	*Staphylococcus sciuri* MarR44	Celery stunt anthracn-ose	*Colletotrichum nymphaeae*	Production of antifungal metabolites (VOCs)	Alijani et al., 2019
*Saccharum officinarum*	*Bacillus subtilis*	Fusarium wilt	*Fusarium* strains	Production of surfactin	Hazarika et al., 2019
*Pisum sativum*	*Pseudomonas chlororaphis*	Black mold, Fusarium wilt	*Aspergillus niger* and *Fusarium oxysporum*	HCN production	Maheshwari et al., 2019a
*Oryza sativa* L.	*Bacillus subtilis*	Bacterial blight of rice, stalk and ear rot, and root rot	*Xanthomonas oryzae, Fusarium verticillioides, Rhizoctonia solani*, and *Sclerotium rolfsii*	Lipopeptide genes encoding surfactin, iturin D, bacillomycin D having antagonistic activities	Kumar V. et al., 2020
*Lilium lancifolium*	*Paenibacillus polymyxa*	Fusarium wilt, gray mold and cankers	*Botryosphaeria dothidea, Fusarium oxysporum, Botrytis cinerea*, and *Fusarium fujikuroi*	Production of antibiotic secondary metabolites	Khan M. S. et al., 2020
*Pennisetum glaucum*	*Bacillus subtilis*	Downy mildew	*Sclerospora graminicola*	Production of siderophore, HCN and ACC deaminase activity.	Sangwan et al., 2021
*Glycine max*	*Bacillus cereus* and *Pseudomonas* sp.	Fusarium wilt	*Fusarium oxysporum, Macrophomina phaseolina*, and *Alternaria alternata*	Production of cellulase, chitinase, and HCN	Dubey et al., 2021
*Helianthus annuus*	*Priestia koreensis*	Fusarium wilt	*Fusarium oxysporum*	Production of essential secondary metabolites and hydrolytic enzymes	Bashir et al., 2021
Disease caused by bacteria					
*Pistacia atlantica* L.	*Pseudomonas protegens*	Bacterial canker	*Pseudomonas syringae* pv. *syringae* Pss20 and *Pseudomonas tolaasii* Pt18	Production of siderophore and protease	Tashi-Oshnoei et al., 2017
*Pyrus communis* L.	Fluorescent *Pseudomonas* sp.	Fire blight disease	*Erwinia amylovora*	Production of antibiotic, PCA, DAPG, pyrrolnitrin and pyoluteorin.	Sharifazizi et al., 2017
*Ventilago madraspatana*	*Enterobacter* sp. CS66	Soft rot and black leg disease	*Pectobacterium atrosepticum*	Quorum quenching	Shastry et al., 2018
*Citrus sinensis*	*Bacillus cereus* Si-Ps1, *Pseudomonas azotoformans* La*-*Pot3*-*3	Bacterial apical necrosis	*Pseudomonas syringae* pv. *syringae* (Pss) B7289 and Pss3289	Quorum quenching	Akbari Kiarood et al., 2020
Disease caused by nematode					
*Musa*	*Streptomyces* sp.	Wilting leaves, gall formation	*Meloidogyne javanica*	Higher abundance of bacterivores	Su et al., 2017
*P. densiflora, P. koraiensis, P. thunbergia, P. rigida*	*Stenotrophomonas* and *Bacillus* sp.	Drying out	*Bursaphelenchus xylophilus*	Production of amocarzine, mebendazole and flubendazole compounds	Shanmugam et al., 2018; Ponpandian et al., 2019
*Fragaria ananassa*	*Bacillus cereus* BCM2	Root-knot disease	*Meloidogyne incognita*	Production of chitosanase, alkaline serine protease, and neutral protease	Hu et al., 2020

*PR, pathogenesis related; VOC, volatile organic compounds; PCA, phenazine-1-carboxylic acid, DAPG: 2,4-diacetyl phloroglucinol.*

## Harnessing “Omics” for Enhancing the Bioactive Metabolites

Bioactive compounds are mostly secondary metabolites produced by the microbes in an active culture cultivation process. Their unique properties have led to lots of research regarding their applications in healthcare as feed supplements, pharmaceuticals, and so on (Singh nee’ Nigam, 2009). Endophytes have been reported to produce many secondary metabolites similar to their host under *in vitro* systems. This ability can lessen our dependency on endangered plants for the extraction of metabolites (Sharma et al., 2021). Several approaches have been employed to harness novel metabolites or to enhance the production of already known ones to support and flourish their large scale application (Du and van Wezel, 2018). Co-culture engineering by culturing more than one type of endophyte together can make us exploit the intermicrobial communications for the enhanced production of bioactive metabolites. A number of studies have reported the production of new metabolites by this method (Stierle et al., 2017; Arora et al., 2018; Gautam et al., 2019). The associated disadvantages such as compatibility issues, competition for substrates, and data acquisition problems pose remarkable challenges (Padmaperuma et al., 2017; Jawed et al., 2019). A number of reports have demonstrated the precise effects of endophytes on the production of secondary metabolites of the host (Khare et al., 2018). Table 5 sums up recent studies dealing with the enhancement of bioactive compounds of the host owing to the endophytes. Other than enhancing the bioactive compounds of the host, endophytes serve as a great treasure of new metabolites which remains largely unexplored looking at the vast diversity of endophytic flora.

**TABLE 5:** Bioactive compounds enhancement in host plants by endophytes.

Host plant	Endophytes	Bioactive compound	Applications	References
*Papaver somniferum*	*Stenotrophomonas maltophilia*	Morphine, Thebaine, Codeine, and Oripavine	Used as analgesics, antitussives and anti- spasmodic	Liscombe and Facchini, 2008; Bonilla et al., 2014
*Aristolochia elegans*	*Piriformospora indica*	Aristolochic acid	Antimicrobial properties	Bagde et al., 2014
*Curcuma longa*	*Azotobacter chroococcum* CL13	Curcumin	Anti-inflammatory, antioxidative, antimalarial activities	Kumar et al., 2016
*Putterlickia verrucosa; Putterlickia retrospinosa*	*Hamigera avellanea*	Maytansine	Cancer chemotherapy	Kusari et al., 2014
*Salvia miltiorrhiza*	*Paecilomyces* sp.	Salvianolic acid	Antioxidative activities	Tang et al., 2014
*Chamomilla recutita* L. Rauschert	*Bacillus subtilis* Co1-6, *Paenibacillus polymyxa* Mc5Re-14	Apigenin-7-*O*-glucoside	Anti-inflammatory capacity	Schmidt et al., 2014
*Panax ginseng*	*Paenibacillus polymyxa.*	Ginsenosides	Anticancerous properties	Gao et al., 2015
*Artemisia annua* L.	*Piriformospora indica* DSM 11827, *Azotobacter chroococcum* W-5	Artemisinin	Artemisinin combination therapies (ACTs) to control malaria	Arora et al., 2016
*Aloe vera*	*Piriformospora indica*	Aloin	Numerous therapeutic applications	Sharma et al., 2014
*Stevia rebaudiana*	*Piriformospora indica*	Enhanced production of Steviol glycosides	High potency sweeteners	Kilam et al., 2017
*Crocus sativus* L.	*Mortierella alpine* CS10E4	Crocin, Picrocrocin, and Safranal	Anti-tumor activities	Wani et al., 2017
*Kadsura angustifolia*	*Umbelopsis dimorpha* SWUKD3.1410	Schitriterpenoids/schinortriterpenoids.	Antihepatitis, antitumor and anti-HIV activities	Qin et al., 2018
*Salvia miltiorrhiza*	*Chaetomium globosum* D38	Phenylpropionic acids and tanshinones	Flavoring agents used in spices (Phenylpropionic acids); Cardiovascular and cerebrovascular protective actions (Tanshinones)	Zhai et al., 2018
*Coleus forskohlii*	*Phialemoniopsis cornearis* SF1, *Macrophomina pseudophaseolina* SF2, *Fusarium redolens* RF1	Davanone, Ethyl cinnamate	Perfumery products, flavoring agents	Mastan et al., 2019

Some approaches have been developed in the recent past to trigger the expression of Biosynthetic gene clusters (BGCs) present in the genome of microbes (endophytes) that can yield some valuable secondary metabolites but remain either silent or poorly expressed. In most cases, BGCs remain silent under laboratory conditions due to complex regulation involved at transcriptional, translational, and post-translational levels. Therefore, the study of changes in gene expression at various levels needs to be done. Recent progress made in bioinformatics, especially genome mining tools have pushed the boundaries of “omics” technologies toward new horizons. It has revolutionized our understanding of the pathways controlling the expression of BGCs.

Genome mining is a powerful approach that can estimate the genetic potential of microbial strain by scanning genomes of interest and identifying the metabolites encoded by BGCs (Ziemert et al., 2016). Whole-genome sequencing and its comparative analysis yield the reconstruction of primary and secondary metabolic pathways that help in suggesting the key metabolic genes to be utilized for metabolic engineering (Palazzotto and Weber, 2018). Metabolic and genetic engineering involves the modulation of biosynthetic enzymes at cellular level/upregulation and downregulation of transcription and translation genes/knock-out and knock-in of desired genes and have been effective in enhancing the production of specific metabolites (Stephens et al., 2015). Mining tools such as antibodies and secondary metabolites analysis shell) (antiSMASH), generalized retro-biosynthetic assembly prediction engine (GRAPE), prediction informatics for secondary metabolomes (PRISM3) have been successful in overcoming the drawbacks associated with manual analyses to some extent. A number of natural products encoded by BGCs remain uncharacterized owing to the complicated regulations occurring at transcriptional, translational, and post-translational levels.

Transcriptome-based studies provide a comparative profile of gene expression and help to assess the key regulators which are in turn used for manifesting the designer strains having the ability to overproduce secondary metabolites (Chaudhary et al., 2013). Proteomics complements other two omics approaches, namely, transcriptomics and genomics, yielding information on differential pathways regulation highlighting key players in the biosynthesis of natural products, which can be used to target for rational engineering (Palazzotto and Weber, 2018). Comparative transcriptomics and proteomics are used to identify the alterations in gene expression associated with the overproduction which are subsequently re-engineered into the organism of interest by considering key genes involved in complex mechanisms. However, its success depends on the reproducibility of the overproducing mechanism in the new target strains (Chaudhary et al., 2013). One of the most important tools of system biology toolbox is metabolomics which catalogs all small metabolites in a biological sample. NMR- and MS-based metabolomic analysis facilitates measurement of low–molecular weight metabolites allowing the metabolic comparison of various biological samples leading to the identification of secondary metabolites from orphan BGCs. A comprehensive picture of metabolic networks helps to engineer the primary metabolism *via* cofactors and precursors for the biosynthesis of any secondary metabolite (Nguyen et al., 2012). Metagenomics is the most commonly used approach to study the chemistry of uncultivated bacteria. It provides a culture-independent approach to exploring the hidden potential of microorganisms.

Omics analysis in isolation is unable to completely unfurl the complexities involved in microbial metabolism associated with the production of secondary metabolites. Therefore, it is necessary to undertake their integration to get better insights into the same (Chaudhary et al., 2013). The combined use of multi-meta-omics approaches such as metabologenomics involves a combination of genome sequencing and automated gene clusters prediction with MS-based metabolomics. It provides us a complete picture of microbial metabolism shedding light on the silent BGCs and the role of natural products (Palazzotto and Weber, 2018). Precision engineering is another modern approach that integrates information from different sources, transcriptome profiling (DNA microarrays), proteome profiling (2D gel electrophoresis), and metabolic profiling (HPLC), thus enabling a more precise identification of key genetic targets and pathways engineered for strain improvement (Gao et al., 2010). Many microbes engineered by metabolic engineering are being used in industrial-scale processes; however, it is associated with challenges such as titers, yields, and productivities required for commercial viability. Different aspects of microbial physiology can also create obstacles for metabolic engineering (Montaño López et al., 2022).

## Limitations and Challenges

Word “endophyte” searched on Google scholar (January 05, 2022) showed 80,100 results indicating extensive research happening in this arena. However, some lacunae need to be filled with regard to the research on endophytes. A sufficient number of studies have not been conducted to study the variations such as plant–microbe interactions on the field induced by a range of environmental and physiological conditions unlike *in vitro*. Information about the synergistic interaction between different microbial taxa such as bacteria, archaea, and fungi, is sparse with most of the studies focusing on each taxa separately.

Although the importance of biofertilizers and biocontrol potential of endophytes over conventional and environment degrading chemical pesticides are well known, some drawbacks of biopesticides are responsible for our slow speed on this eco-friendly path. High production cost and limited period of activity as compared to the chemical ones along with lower potency make it difficult for the farmers to opt for it. Owing to their target-specific nature, they control a specific portion of pests in the treated area and may leave the other damage-causing pests unaffected (Kawalekar, 2013). Lesser insights are available into the overlaps present in the metabolic pathways of endophytes and host plants, which leads to the production of a particular bioactive compound (Mishra et al., 2021). More research targeted at unfurling the genetic controls involved in stress tolerance conferring potential as well as bioactive compounds accumulation capability of the microbe is to be undertaken to unfold the molecular mechanisms behind the same.

## Conclusion

Plant and endophytes exist in close interaction with each other and provide increased productivity as a bonus. The effects and functions of these associations have not been understood fully thereby calling for a more in-depth study. It would be more beneficial if more knowledge of endophytes’ ecology and their molecular interaction is made available for harnessing and application in agriculture. This research will have a progressive impact on the environment in the direction of chemical fertilizer–free cultivation and better contribution to the economy. Also, the optimization of growth conditions as well as nutrient media for the endophytes having enormous potential to be applied particularly in pharmaceutical and agricultural sectors needs to be done at the earliest. “Omics” combined with recent computational data mining tools can help unravel the functions of complex plant microbiome, which can provide us with more competent microbes as far as stress tolerance and enhancing the bioactive metabolites is concerned.

## Author Contributions

PS and AD conceptualized the theme of this review. SR wrote and compiled the original draft. PK, PD, and RM drafted the figures and compiled tables. All authors have made intellectual and substantial contribution and approved it for publication.

## Conflict of Interest

The authors declare that the research was conducted in the absence of any commercial or financial relationships that could be construed as a potential conflict of interest.

## Publisher’s Note

All claims expressed in this article are solely those of the authors and do not necessarily represent those of their affiliated organizations, or those of the publisher, the editors and the reviewers. Any product that may be evaluated in this article, or claim that may be made by its manufacturer, is not guaranteed or endorsed by the publisher.
